# Trastuzumab deruxtecan in advanced breast cancer: a real-world study of efficacy and safety in Chinese cohort with HER2-positive and HER2-low expression

**DOI:** 10.3389/fonc.2026.1758757

**Published:** 2026-03-16

**Authors:** Hainan Liang, Zhengqiu Zhu, Feifei Kong

**Affiliations:** Department of Oncology, Affiliated Hospital of Xuzhou Medical University, Xuzhou, Jiangsu, China

**Keywords:** advanced breast cancer, HER2, real-world study, T-DXd, trastuzumab deruxtecan

## Abstract

**Objective:**

To explore the safety and real-world efficacy of trastuzumab deruxtecan in Chinese patients with advanced breast cancer that is either HER2-positive or HER2-low expression.

**Methods:**

A retrospective analysis was conducted on 104 patients diagnosed with advanced breast cancer who received trastuzumab deruxtecan treatment at the Affiliated Hospital of Xuzhou Medical University, with the study period spanning from January 2023 to September 2025. The primary endpoint of the study was progression-free survival, and the secondary endpoints were objective response rate, disease control rate, and safety. Progression-free survival was analyzed using the Kaplan-Meier method and log-rank test.

**Results:**

Among 104 patients, the median progression-free survival was 8.8 months in those with HER2-low expression, whereas HER2-positive patients exhibited a longer median progression-free survival of 14.2 months, along with superior objective response rate and disease control rate. Adverse events were reported in all patients, predominantly Grade I-II, with no new safety signals detected.

**Conclusions:**

Consistent with findings from previous clinical trials, real-world evidence from this study confirms that trastuzumab deruxtecan exhibits efficacy in patients with advanced breast cancer across all HER2 expression statuses and safety profile is manageable. Earlier initiation of trastuzumab deruxtecan may maximize survival benefits. These findings help bridge the gap between clinical trial evidence and routine clinical practice.

## 1 Introduction

Breast cancer (BC), characterized by the malignant proliferation of cells originating primarily from mammary ducts or lobules, represents the most commonly diagnosed cancer among women worldwide (1, 2). Around 30% of BC patients experience progression to advanced disease throughout their clinical course, those with advanced breast cancer (ABC) face lower 5-year survival rates compared to individuals with early-stage disease, highlighting the imperative for effective therapeutic strategies (3).

The clinical management of BC is challenged by its significant molecular heterogeneity (4). The human epidermal growth factor receptor 2 (HER2) molecule is an independent predictor factor for poor outcomes in BC (5). This alteration occurs in roughly 20%–30% of BC cases and is identified by immunohistochemistry (IHC) 3+ or 2+ with positive fluorescence *in situ* hybridization (FISH). This molecular alteration is associated with high tumor invasiveness, rapid disease progression, increased risk of recurrence, and unfavorable prognosis (6). Although the use of anti-HER2 targeted agents such as trastuzumab and pertuzumab have significantly improved survival outcomes, many patients still experience either intrinsic or acquired resistance during treatment, leading to disease progression (7). Additionally, approximately 45%-55% of BC patients belong to the HER2-low expression subgroup, defined as those with an IHC score of 1+ or 2+ combined with negative FISH findings (8). This population, previously lacking targeted options, often received heterogeneous treatments with suboptimal outcomes.

The recent advancement in the development of antibody-drug conjugates (ADCs), which deliver potent cytotoxic payloads specifically to tumor cells, has revolutionized this landscape (9). Trastuzumab deruxtecan (T-DXd) is a novel HER2-directed ADC with a topoisomerase I inhibitor as its payload. Additionally, T-DXd has a drug-to-antibody ratio (DAR) of 8:1, which is significantly higher than that of other ADCs, further enhancing its antitumor efficacy (10). Pivotal clinical trials, including DESTINY-Breast03 (DB-03) and DB-04, have established the robust efficacy of T-DXd in heavily pretreated HER2-positive and HER2-low ABC, showing significant improvements in objective response rate (ORR) and progression-free survival (PFS) (10, 11). Notably, while trastuzumab-based therapies have markedly improved outcomes for HER2-positive metastatic breast cancer, T-DXd has demonstrated even more substantial survival benefits in later-line settings. Remarkably, the survival expectations observed with T-DXd in these heavily pretreated HER2-positive patients may approach or even exceed those historically seen in the typically more indolent hormone receptor-positive (HR+) breast cancer subgroup, underscoring its transformative potential. More recently, the DB-06 trial further expanded the therapeutic landscape by demonstrating significant efficacy of T-DXd in patients with HR+, HER2-low, and notably, HER2-ultralow (formally defined as IHC 0+ or IHC 1+) metastatic breast cancer who had progressed on prior endocrine therapy combined with a CDK4/6 inhibitor (12).

However, T-DXd’s real-world data on its efficacy and safety in Chinese populations remain sparse. Clinical trial results, while robust, reflect efficacy under ideal conditions, and their generalizability to real-world populations requires verification. Against this backdrop, this study aims to investigate the real-world application status, efficacy, and safety of T-DXd in patients with ABC in China. By bridging the gap between clinical trial data and real-world practice, it seeks to inform and refine treatment decision-making in diverse clinical settings.

## 2 Materials and methods

### 2.1 Study design and patients

**Figure 1:**
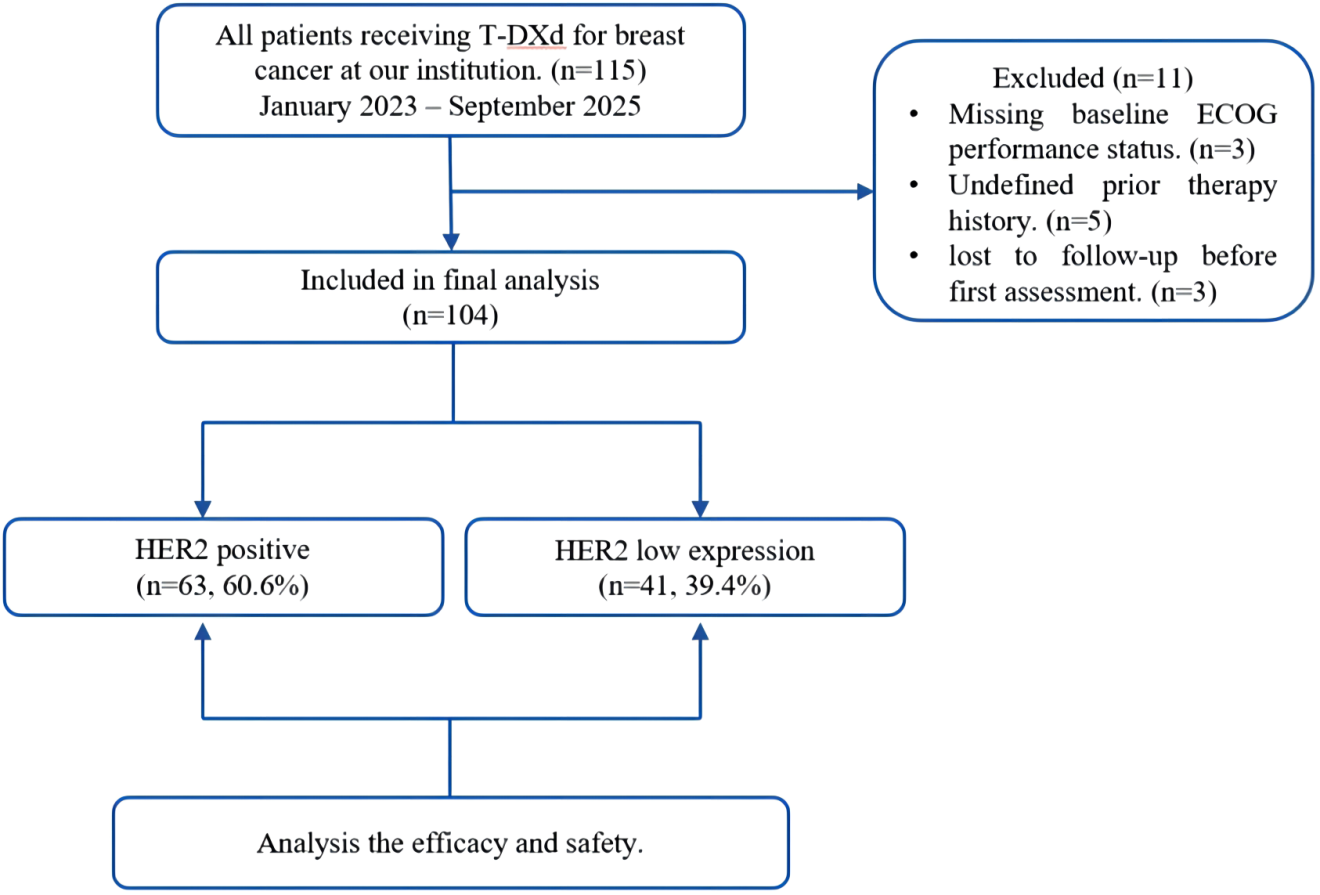
Flow diagram.

A total of 104 patients diagnosed with ABC, including those with HER2-positive or HER2-low expression, who underwent T-DXd treatment at the Affiliated Hospital of Xuzhou Medical University between January 2023 and September 2025 were included in the analysis. HER2 positivity was defined as IHC 3+ or 2+ with positive FISH. HER2-low expression was defined as IHC 1+ or 2+ with negative FISH (13). For patients who underwent multiple biopsies during the course of their illness, the HER2 expression status used for cohort grouping and all subsequent analyzes was determined based on the biopsy sample obtained closest to the initiation of T-DXd treatment. The inclusion criteria for this study are as follows: Aged >= 18 years; Pathologically confirmed HER2-positive or HER2-low expression BC; Imaging-confirmed unresectable or metastatic ABC; Patients with HER2-positive disease had received at least one prior anti-HER2 therapy, whereas those with HER2-low expression experienced disease recurrence within 6 months of adjuvant chemotherapy or had undergone at least one course of systemic therapy for metastatic disease; Presence of measurable lesions in accordance with the RECIST 1.1; Available and complete medical records during previous treatment. Patients were excluded if they met any of the following criteria: concurrent diagnosis of other malignant tumors; complicated by severe non-tumor-related diseases involving other organ systems; being pregnant or lactating women; or having incomplete clinical data, which precluded confirmation of their pre-treatment ECOG performance status and therapeutic history. The detailed flow diagram is shown in **Figure 1**.

### 2.2 Treatment methods and efficacy and safety assessment

T-DXd was initially administered at a dose of 5.4 mg/kg on Day 1 of each treatment cycle, with one cycle defined as 21 days. The course of treatment was maintained until the disease progressed or unbearable adverse events (AEs) occurred. In the event of AEs, dose adjustments or modifications to the administration regimen were made in accordance with the drug package insert and the physician’s clinical judgment.

All patients underwent baseline hematological and imaging assessments prior to treatment initiation to comprehensively assess their current disease status. Efficacy was reassessed every two treatment cycles by investigator assessment according to RECIST 1.1, and categorized into four outcomes including complete response (CR), partial response (PR), stable disease (SD), and progressive disease (PD). Two crucial efficacy endpoints were determined: ORR is the percentage of patients whose best overall tumor response was either CR or PR; disease control rate (DCR) is the percentage of patients whose best overall tumor response was either CR, PR, or SD. Routine hematological examinations including blood routine, liver function and renal function tests were repeated weekly. AEs in patients were evaluated and graded according to the NCI-CTCAE 5.0, with grades ranging from Grade I to Grade IV.

Given the known treatment-related risks of T-DXd, an enhanced monitoring program was implemented for interstitial lung disease (ILD) and cardiac dysfunction. All patients enrolled in the study underwent systematic baseline data assessment before treatment, including baseline chest imaging scans, complete pulmonary function tests, detailed respiratory system history collection, and cardiac echocardiography and comprehensive cardiac function assessment. Imaging and cardiac ultrasound examinations were repeated every 6–8 weeks.

### 2.3 Follow-up

Patients were followed up by reviewing inpatient medical records, outpatient visit documentation, and telephone follow-up calls. The last follow-up date was September 2025. Follow-up was discontinued for patients who exhibited disease progression, died, or were lost to follow-up. PFS was defined as the interval from the start of T-DXd administration to the first occurrence of PD, death, or the date of the final follow-up visit.

### 2.4 Statistical methods

Statistical analyses for all patients were performed using SPSS (Version 29.0) and R (version 4.3.1). PFS was estimated using the Kaplan-Meier method, and the log-rank test was applied for survival outcomes. Intergroup comparisons were carried out using the χ² test and Fisher’s exact test for categorical variables, depending on the characteristics of the data. Association between clinicopathological characteristics and survival outcomes was assessed using Cox regression analysis, variables with P<0.05 in univariate analysis were included in the multivariate Cox proportional hazards regression model, and independent prognostic factors were screened by stepwise regression method, and the hazard ratio (HR) and its 95% Confidence Interval (95% CI) were calculated and reported. For the Cox proportional hazards regression models, the former group was designated as the reference category for two-group comparisons. P<0.05 was indicative of statistical significance.

## 3 Results

### 3.1 Patients’ characteristics

The baseline characteristics of the 104 patients are summarized in **Table 1**. All of the patients, 63 of whom had HER2-positive disease and 41 of whom had HER2-low expression, had pathologically proven BC. A higher proportion of patients in the HER2-low expression group were HR-positive (80.5%) compared to the HER2-positive group (61.9%). HR- was defined as negative for both estrogen receptor (ER) and progesterone receptor (PR), whereas HR+ was defined as positive for either ER or PR, or both (14). All patients in both groups had metastases of varying severity, brain metastases were present in 27.0% of HER2-positive and 24.4% of HER2-low expression patients. With respect to T-DXd treatment lines, 46(44.2%) patients received post-third-line treatment. And all HER2-positive patients had prior anti-HER2 exposure, and all HR+ patients in the HER2-low group had prior endocrine-based therapy.

**Table 1:** Baseline clinical and pathological characteristics of patients stratified by HER2 expression status.

Characteristic	HER2-positive (n=63, %)	HER2-low expression (n=41, %)	Total population (n=104)
Age			
Median(years)	55	56	55
Range(years)	31-69	35-78	31-78
ECOG			
0-1	49(77.8)	29 (70.7)	79(76.0)
>=2	13(20.6)	12 (29.3)	25(24.0)
Ki-67 expression			
>30%	40(63.5)	24(58.5)	64(61.5)
<=30	23(36.5)	17(41.5)	40(38.5)
HER2 status			
IHC3+	46(73.0)	0	46(44.2)
IHC2+/FISH+	17(27.0)	0	17(16.3)
IHC2+/FISH-	0	26(63.4)	26(25.0)
IHC1+	0	15(36.6)	15(14.4)
HR status			
Negative ER and PR	24(38.1)	8 (19.5)	32(30.8)
Positive ER and/or positive PR	39(61.9)	33(80.5)	72(69.2)
Past medical history			
With chronic disease	16(25.4)	13(31.7)	29(27.9)
Without chronic disease	47(74.6)	28(68.3)	75(72.1)
Menopausal status			
Postmenopausal	44(69.8)	28(68.3)	72(69.2)
Premenopausal	19(30.2)	13(31.7)	32(30.8)
Histological Type			
Invasive ductal carcinoma	47(74.6)	29(70.7)	76(73.1)
Other types	16(25.4)	12(29.3)	28(26.9)
Metastatic site			
Brain metastasis	17(27.0)	10(24.4)	27(26.0)
Lung metastasis	10(15.9)	13(31.7)	23(22.1)
Liver metastasis	26(41.2)	18(43.9)	44(42.3)
Bone metastasis	26(41.2)	20(48.8)	46(44.2)
Lymph node metastasis			
Yes	43(68.3)	28(68.3)	71(68.3)
No	20(31.7)	13(31.7)	33(31.7)
Radiotherapy history			
Yes	38(60.3)	10(24.4)	48(46.2)
No	25(39.7)	31(75.6)	56(53.8)
Prior treatment lines			
<=3 lines	39(61.9)	19(46.3)	58(55.8)
>3 lines	24(38.1)	22(53.7)	46(44.2)
Prior anti-HER2 therapy			
Trastuzumab	63(100%)	0	63(60.6)
Pertuzumab	56(88.9)	0	56(53.8)
Any ADCs	18(28.6)	0	18(17.3)
Prior CDK4/6 inhibitor			
Yes	4(6.3)	33(80.5)	37(35.6)
No	59(93.7)	8 (19.5)	67(64.4)

ECOG, Eastern Cooperation Oncology Group; Ki-67, Ki-67 Index; IHC, Immunohistochemistry; FISH, Fluorescence in Situ Hybridization; HR, Hormone Receptor; ER, Estrogen Receptor; PR, Progesterone Receptor.

### 3.2 Clinical efficacy

**Figure 2:**
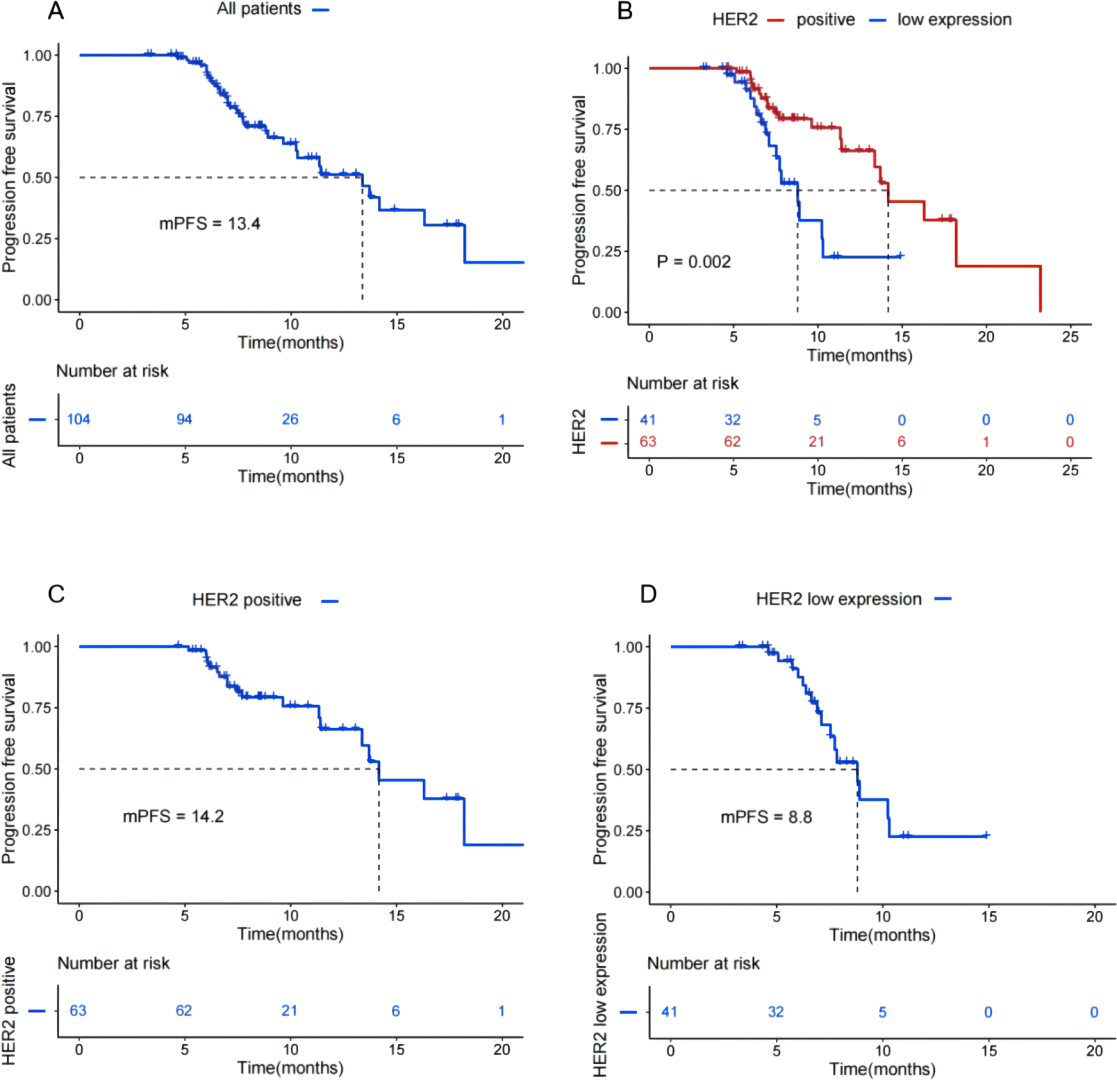
Kaplan-Meier survival curves for PFS in patients treated with T-DXd, **(A)** Kaplan-Meier curve for PFS in All patients treated with T-DXd, **(B)** Kaplan-Meier curve for PFS in HER2- positive subgroup vs in HER2-low expression subgroup. **(C)** Kaplan-Meier curve for PFS in HER2-positive patients. **(D)** Kaplan-Meier curve for PFS in HER2-low expression patients.

The median follow-up duration was 8.57 months (range, 3.23-23.20), estimated using the reverse Kaplan-Meier method. At the time of data analysis, a total of 36 (34.6%) disease progression or death events were observed in 104 patients, and 68 (65.4%) were still receiving T-DXd therapy at the time of data acquisition. As shown in **Figure 2**, given the immature follow-up data, the estimated results indicated that HER2-positive subgroup’s median PFS (mPFS) was 14.2 months (95% CI: 11.115-17.225), and the 12-month PFS rate was 66.2%. In contrast, the mPFS was 8.8 months (95% CI: 7.515-10.085) in the HER2-low expression subgroup, and the 12-month PFS rate was 22.6%. Longer mPFS was observed in HER2-positive patients versus those with low HER2 expression (*P* = 0.002). This finding indicates a significant association between HER2 expression level and the survival benefit derived from T-DXd treatment. **Table 2** illustrates that, all patients were evaluable for efficacy, in HER2-positive group, the ORR was 30.2% (95%CI: 19.3%-43.1%) and the DCR was 68.3% (95%CI: 55.4%-79.5%). In HER2-low expression group, the ORR was 19.5% (95%CI: 8.8%-34.9%) and the DCR was 61.0% (95%CI: 44.5%-75.8%). It can be seen that HER2-positive patients have higher ORR and DCR than HER2-low expression patients.

**Table 2:** Overall response.

	HER2-positive (n=63, %)	HER2-low expression (n=41, %)
CR	3(4.8)	0
PR	16(25.4)	8(19.5)
SD	24(38.1)	17(41.5)
PD	20(31.7)	16(39.0)
ORR	19(30.2)	8(19.5)
95% CI	19.3%, 43.1%	8.8%, 34.9%
DCR	43(68.3)	25(61.0)
95% CI	55.4%, 79.5%	44.5%, 75.8%

CR, Complete Response; PR, Partial Response; SD, Stable Disease; PD, Progression Disease; ORR, Objective Response Rate; DCR, Disease Control Rate; 95%CI, Confidence Interval.

As **Table 3** illustrates, comparison of patients enrolled in this study with 261 HER2-positive patients from the DB-03 trial, as well as 373 HER2-low expression patients in the T-DXd treatment arm of the DB-04 trial, revealed that patients receiving T-DXd had more advanced disease stages and a heavier comorbidity burden (10, 11). However, more than half of the patients in the HER2-positive group continued to be free of disease progression, and their mPFS continued to reach 14.2 months. The mPFS for HER2-low expression group was 8.8 months. These findings show that although the efficacy in real-world settings is lower than that observed in clinical trials, most ABC patients who have undergone multiple prior lines of treatment can still derive significant survival benefits from T-DXd.

**Table 3:** Baseline characteristics: this study vs. DB-03 trial and DB-04 trial.

Baseline characteristic	HER2-positive (n=63)	HER2-low expression (n=41)	DESTINY-breast 03 trial (n=261)	DESTINY-breast 04 trial (n=373)
Median age (years)	55(31-69)	56(28-81)	54(28-81)	57(31-81)
ECOG				
0-1	49(77.8%)	29 (70.7%)	100%	100%
>=2	13(20.6%)	12(29.3%)		
HR+	39(61.9%)	33(80.5%)	50.2%	88.7%
Brain metastasis	17(27.0%)	10(24.4%)	16.5%	5.4%
Prior treatment lines > 3	24(38.1%)	22(53.7%)	18.4%	24.0%
mPFS	14.2m.	8.8m.	28.8m.	10.1m.
ORR	30.2%	19.5%	79.0%	57.3%
DCR	68.3%	61.0%	96.0%	91.2%

ECOG, Eastern Cooperation Oncology Group; HR, Hormone Receptor; mPFS, median Progression-free Survival.

### 3.3 Subgroup analysis

Further subgroup analyses evaluated the clinical factors that potentially influence the efficacy of T-DXd. **Table 4** summarizes the results of multivariate Cox regression analyses for PFS. Multivariate Cox regression analysis identified ECOG performance status (>=2 vs.0–1 HR = 2.801, 95%CI=1.402-5.598), the number of lines of prior therapy (<=3 lines vs.>3 HR = 0.320, 95%CI=0.149-0.688), and the HER2 expression status (positive vs. low expression HR = 0.349, 95%CI=0.172-0.708) as independent predictor factors for therapeutic response to T-DXd in patients with ABC (all P<0.05).

**Table 4:** Univariate analysis of factors associated with mPFS in all patients.

Factor	Univariate			Multivariate		
	HR	95%CI	*P* value	HR	95%CI	*P* value
Age(>=65or<65)	1.083	0.417–2.815	0.869			
ECOG (2or 0-1)	2.888	1.459–5.718	0.002	2.801	1.402–5.598	0.004
Ki-67 Index (>=30%or<30%)	1.137	0.565–2.290	0.719			
HR Status (+or-)	1.027	0.516–2.045	0.940			
Menopausal Status (Yes or No)	0.760	0.360–1.605	0.472			
Brain Metastasis (Yes or No)	0.570	0.263–1.233	0.153			
Lung Metastasis (Yes or No)	1.351	0.655–2.787	0.416			
Liver metastasis (Yes or No)	0.929	0.464–1.863	0.873			
Bone metastasis (Yes or No)	1.352	0.693–2.636	0.377			
Lymph Node Metastasis (Yes or No)	1.339	0.638–2.809	0.440			
Number of Treatment Lines (<=3or>3)	0.316	0.151–0.661	0.002	0.320	0.149–0.688	0.004
HER2 Expression Status (+ or -)	0.349	0.172-0.708	0.004	0.372	0.179-0.774	0.008

ECOG, Eastern Cooperation Oncology Group; Ki-67, Ki-67 Index; HR, Hormone Receptor; 95%CI: Confidence Interval.

**Figure 3:**
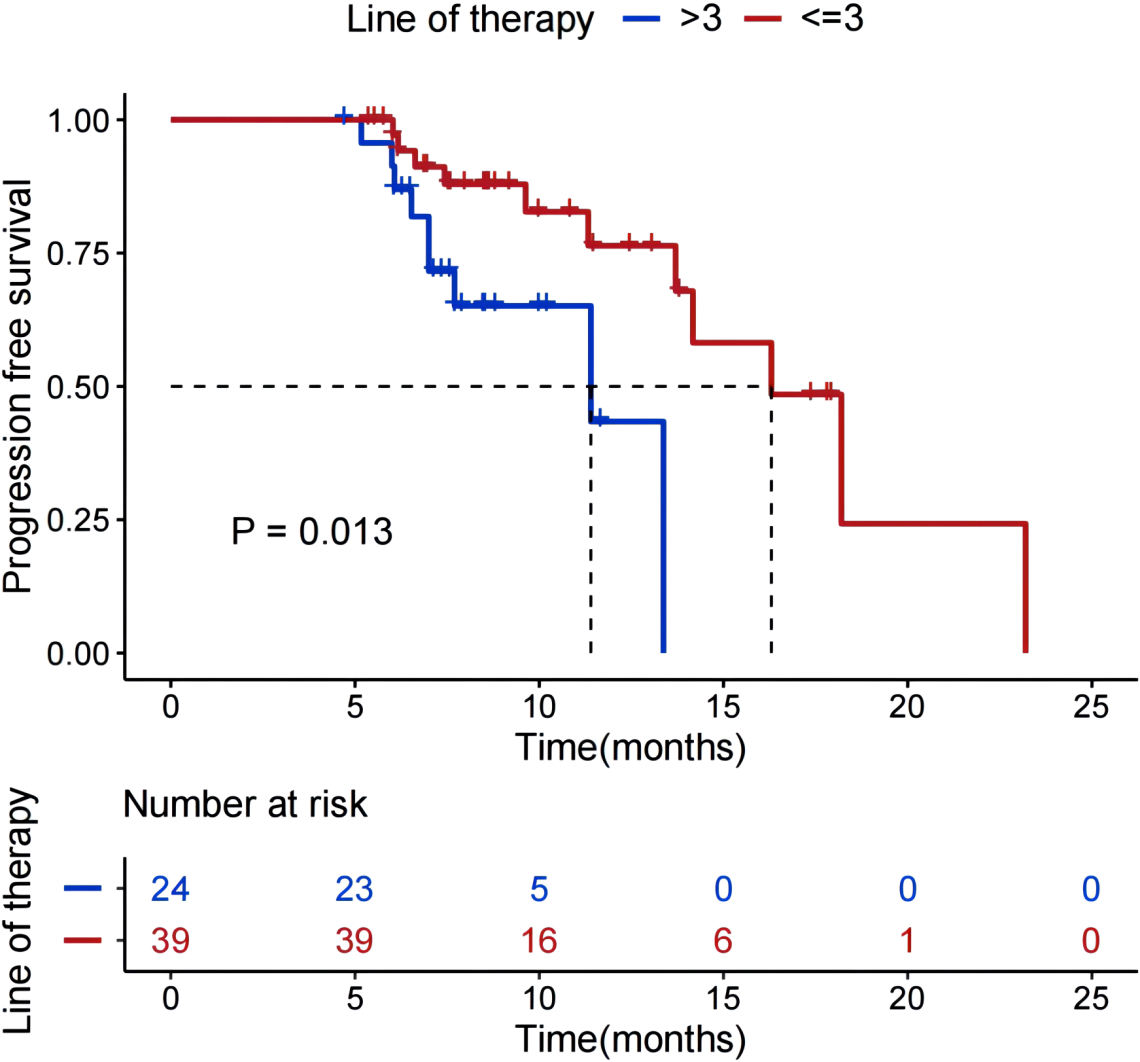
Kaplan-Meier survival curves for PFS in HER2-positive patients stratified by the number of prior treatment lines (<=3 vs >3 lines).

**Figure 4:**
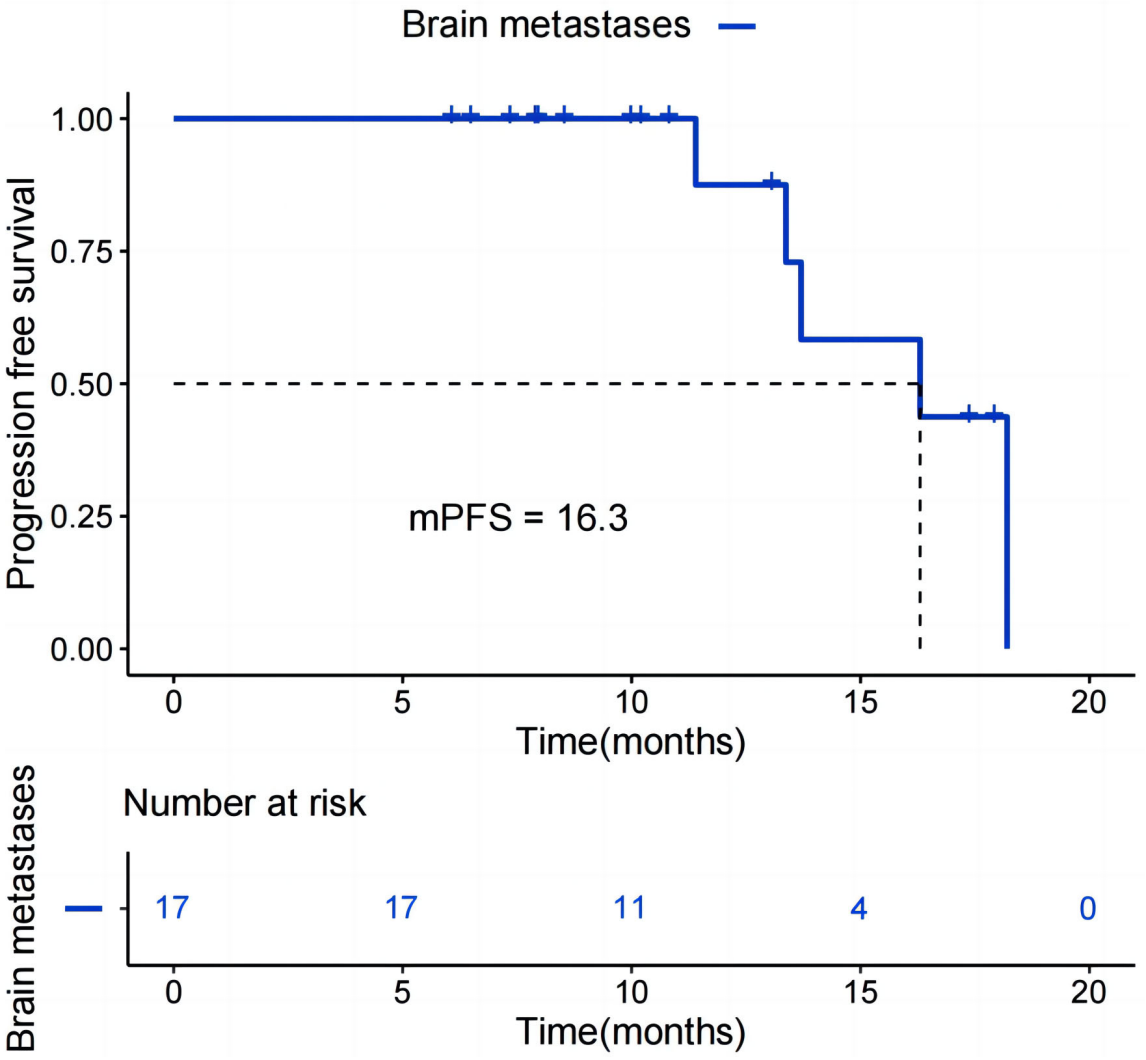
Kaplan-Meier survival curves for PFS in patients with BM among the HER2-positive subgroup.

As shown in **Figure 3**, stratified analysis by the number of prior treatment lines revealed that patients who received<=3 lines of prior therapy had a mPFS of 16.3 months, which was significantly superior to the 11.4 months observed in patients who received >3 lines of prior therapy (P = 0.013). As can be seen from **Figure 4**, in the HER2-positive subgroup enrolled in this study, 17 patients with BM achieved mPFS of 16.3 months, and the CR rate was 17.6%. All patients in the HER2-positive subgroup had received prior anti-HER2 treatment.

### 3.4 Safety

As shown in **Table 5**, all 104 patients in this cohort experienced AEs of varying severity, and the AEs were characterized by involvement of multiple systems. Within the hematological system, neutropenia was the most prominent AEs. Among HER2-positive group, 27 cases (42.9%) developed this symptom, of which 4 cases (6.3%) were classified as grade III-IV. Among HER2-low expression group, 14 cases (34.1%) patients experienced neutropenia, with 3 cases (7.3%) being grade III-IV. Additionally, anemia, leukopenia, and thrombocytopenia also occurred at notable rates, however, less than 10% of patients experienced grade III-IV AEs for these conditions. The incidence of digestive system AEs was relatively high. However, most were grade I–II, and only a small proportion of patients developed liver function impairment. ILD is a relatively severe and clinically concerning adverse event (AE) linked to T-DXd treatment, and its management is complex (15). In this study, among both HER2-positive and HER2-low expression subgroup, only 1 patient in each subgroup developed grade I ILD. All these patients showed clinical improvement following T-DXd discontinuation and treatment. One patient in the HER2 low expression subgroup experienced a decrease in left ventricular ejection fraction (LVEF). Cardiac ultrasound showed that the ejection fraction decreased by >10% compared with the baseline. At the same time, the patient was observed to have lower limb edema symptoms, and symptomatic treatment was given and T-DXd treatment was subsequently stopped. There was no significant difference in the incidence of AEs between the two groups (all *P*>0.05).

**Table 5:** Summary of adverse events.

AEs term	HER2-positive (n=63, %)			HER2-low expression (n=41, %)			*P* value
	Grade I-II	Grade III-IV	Total	Grade I-II	Grade III-IV	Total	
Hematological system							
Leukopenia	15(23.8)	3 (4.8)	18(28.6)	13 (31.7)	2 (4.9)	14(34.1)	0.547
Neutropenia	23(36.6)	4 (6.3)	27(42.9)	12(29.3)	3 (7.3)	14(34.1)	0.466
Anemia	18(28.6)	4 (6.3)	22(36.1)	14(34.1)	1 (2.4)	15(36.9)	0.862
Thrombocytopenia	14(22.2)	3 (4.8)	17(27.0)	9 (22.0)	2 (4.9)	11(26.8)	0.986
Digestive system							
Nausea	49(77.8)	0	49(77.8)	33(80.5)	0	33(80.5)	0.741
Vomiting	27(42.9)	0	27(42.9)	17(41.5)	0	17(41.5)	0.888
Diarrhea	10(15.9)	0	10(15.9)	10(24.4)	0	10(24.4)	0.281
ALT/AST elevation	16(25.4)	3 (4.8)	19(30.2)	10(24.4)	3 (7.3)	13 (31.7)	0.867
ALP/GGT elevation	14(22.2)	1 (1.6)	15(23.8)	9 (22.0)	2 (4.9)	11(26.8)	0.728
Anorexia	49(77.8)	0	49(77.8)	33(80.5)	0	33(80.5)	0.741
Respiratory system							
ILD	1 (1.6)	0	1 (1.6)	1 (2.4)	0	1 (2.4)	1.000
Cardiovascular system							
LVEF Decrease	0	0	0	1 (2.4)	0	1 (2.4)	0.394
Systemic symptoms							
Fatigue	35(55.6)	0	35(55.6)	25(61.0)	0	25(61.0)	0.585
Pain	19(30.2)	2 (3.2)	21(33.3)	14(34.1)	0	14(34.1)	0.932
Edema	6 (9.5)	0	6 (9.5)	4 (9.8)	0	4 (9.8)	1.000

AEs, Adverse Events; ILD, Interstitial Lung Disease; ALT, Alanine Aminotran Sferase; AST, Aspartate Aminotran Sferase; ALP, Alkaline Phosphatase; GGT, Gamma-Glutamyl Transferase; LVEF, left ventricular ejection fraction.

## 4 Discussion

Excellent efficacy of T-DXd in HER2-positive ABC patients who previously received multiple-line therapy has been indicated in the DB-02 and DB-03 clinical trials, and DB-04 has shown that the beneficiary population includes those with HER2-low expression (10, 11, 16). Accordingly, T-DXd has been approved in China for HER2-positive ABC after at least one previous anti-HER2 regimen, and for HER2-low expression disease following prior systemic therapy in the metastatic context or recurrence during or within 6 months of completing adjuvant chemotherapy (17). More recently, the phase III DB-06 shown that T-DXd significantly prolonged PFS when compared to conventional chemotherapy in HR-positive, HER2-low, or ultra-low expression patients who progressed despite previous regimens with endocrine therapy combination with CDK4/6 inhibitors (12). Consistently, the DB series have confirmed that T-DXd significantly providing PFS and OS, offering new treatment to patients with ABC. However, the efficacy demonstrated in these pivotal trials, conducted under standardized protocols, may not fully translate to effectiveness in real-world clinical practice. Notably, although Chinese patients were included in both the DB-03 and DB-04 trials, only 58.2% and 38.2% of the cohorts were Asian patients, respectively, with the proportion of Chinese patients within these Asian subgroups was unreported (18). Importantly, due to differences in genetic backgrounds and clinical practices, the actual clinical treatment patterns and baseline features of Chinese ABC patients may differ from those of Western populations (19). Thus, this study focuses on Chinese patients with ABC, and its findings demonstrate that T-DXd exhibits favorable efficacy regardless of HER2 expression levels, providing targeted evidence for clinical decision-making in Chinese patients.

### 4.1 Efficacy analysis

This study confirms that T-DXd exhibits significant therapeutic efficacy even in patients with poor prognosis, such as those undergoing advanced-line treatment or complicated with BM, albeit with notable efficacy heterogeneity across different patient subgroups. Our analysis revealed that HER2-positive patients achieved an mPFS of 14.2 months, with significantly superior clinical benefits compared to HER2-low expression patients who had an mPFS of 8.8 months. This efficacy difference is highly consistent with the core findings of the DB-03 trial, which reported an mPFS of 28.8 months in HER2-positive patients, and the DB-04 trial, which reported an mPFS of 10.1 months in HER2-low expression patients (10, 11). Although the real-world efficacy of T-DXd in this study was slightly lower than that in the DB series of clinical trials. Our findings from another prospective phase II DAISY trial conducted in France indicated that HER2 expression level is a key determinant of T-DXd efficacy (20). The trial’s data showed that HER2-positive patients (n=72) had an mPFS of 11.1 months, while HER2-low expression patients (n=40) had an mPFS of 6.7 months. Additionally, a multicenter, observational real-world study conducted in China demonstrated that the mPFS was 10.51 months among HER2-positive patients (n=32) and 10.18 months among HER2-low expression patients (n=29) (21). These two studies’ findings were highly consistent with the results of the present study, and our study’s findings even outperformed those of these two trials. Notably, 80.5% of patients in the HER2-low cohort were HR-positive in the present study, compared with 61.9% of those in the HER2-positive cohort. Additionally, the HER2-low group had a markedly higher proportion of patients with more than three prior lines of therapy than the HER2-positive group (53.7% vs. 38.1%). To disentangle the independent effect of HER2 expression from the potential confounding influences of HR status and prior treatment line number, both variables were incorporated into our multivariate regression model. After adjustment for HR status and treatment lines, HER2 expression was still significantly correlated with PFS, which further supported the conclusion that HER2 expression level is an independent predictor of T-DXd efficacy, thus confirming that HER2 expression level is a key determinant of T-DXd efficacy across diverse populations and clinical settings.

Further subgroup analysis of HER2-positive patients revealed that patients who received <=3 lines of prior therapy had a significantly longer mPFS (16.3 months) than those with >3 lines (11.4 months, P = 0.013). And multivariate Cox regression revealed a significant association between the efficacy of T-DXd and the number of prior treatment lines (<=3 lines vs.>3 HR = 0.320, 95%CI=0.149–0.688, *P* < 0.05). This finding is consistent with the DB-02 trial, which also reported a relatively shorter mPFS in heavily pretreated patients receiving T-DXd (16), collectively underscoring that the depth of prior treatment lines may negatively impact T-DXd efficacy. These data suggest that earlier administration of T-DXd might be critical to maximizing patient benefit. This premise is strongly supported by the phase III DB-09 trial, which demonstrated that first-line T-DXd significantly prolonged mPFS, improved objective and complete response rates, and had a manageable safety profile compared to the standard THP (trastuzumab, pertuzumab, and taxane) regimen in patients with HER2-positive ABC (22). Therefore, our results, combined with evidence from pivotal trials, position T-DXd as a compelling early-line treatment option to optimize outcomes in HER2-positive ABC.

### 4.2 Safety analysis

All 104 patients evaluated for safety in this study experienced AEs involving multiple organ systems. of varying severity, with the adverse reactions characterized by involvement of multiple systems. Gastrointestinal toxicities were most frequently reported, with anorexia, nausea, and vomiting being the most common manifestations. These AEs were primarily Grade I-II in severity, predominantly occurred at treatment initiation, and generally ameliorated as therapy continued, indicating that most patients developed mild tolerance. Hematological AEs were also observed with a relatively high incidence, with a low rate of Grade III-IV events, underscoring the need to monitor bone marrow function. Although hepatic AEs were less common, a subset of patients experienced Grade III-IV elevations in aspartate transaminase (AST), alanine transaminase (ALT), alkaline phosphatase (ALP), and gamma-glutamyl transferase (GGT), necessitating enhanced liver function monitoring during treatment. Notably, the incidence of drug-induced interstitial lung disease (ILD) was low in our cohort, which is a well-recognized and serious AE associated with T-DXd (15). Among the 104 enrolled patients who underwent rigorous baseline and on-treatment pulmonary monitoring, only two (1.9%) developed Grade I ILD. Both cases improved after treatment interruption and corticosteroid administration. Although the incidence of ILD was low in this study, a high level of vigilance remains necessary when T-DXd is used in clinical practice.

The AE profile observed in our study aligns with, yet shows some distinctions from, that reported in pivotal trials. This is consistent with the findings reported in the DB-04 trial, compared with chemotherapy, T-DXd associated hematological AEs including leukopenia and neutropenia and hepatic AEs including elevated aminotransferases were less severe (11). This finding may be associated with T-DXd’s precise release of toxins within tumor cells, which reduces the concentration of chemotherapeutic agents in the systemic circulation (8). In contrast, gastrointestinal reactions including nausea and vomiting were more severe, potentially due to the toxicity of the topoisomerase I inhibitor payload it carries (23).

### 4.3 Clinical relevance

In patients with HER2-positive metastatic BC, 30%-50% present with BM (24). BM remains a core challenge in conventional treatment, as most chemotherapeutic agents and even some targeted therapies (e.g., trastuzumab) fail to penetrate the blood-brain barrier effectively. This inadequate penetration results in suboptimal drug concentrations within the brain, leading to limited therapeutic efficacy (25). T-DXd can cross the blood-brain barrier or indirectly enter the brain via disruption of the tumor microenvironment, and it accumulates locally in BM lesions. It then releases its cytotoxic payload to directly eliminate HER2-positive tumor cells within the brain (26). The DB-03 trial reported that among HER2-positive patients with BM, the mPFS was 14.1 months (10). Among the HER2-positive patients enrolled in this study, 17 had BM. For this subgroup, the mPFS was 16.3 months, which were comparable to the results of the DB-03 trial. The phase IIIb/IV DB-12 clinical trial demonstrated that among HER2-positive ABC patients with disease progression after receiving ≤2 lines of treatment in the metastatic setting, the mPFS was 17.3 months in the BM cohort (27). Although the results of this study were slightly lower than those reported in DB-12 clinical trials, it is important to note that 9 out of 17 patients with BM in this subgroup received later-line treatment. Patients receiving later-line therapy typically exhibit stronger drug resistance, leading to generally limited therapeutic responses, which may be one of the reasons for the relatively lower efficacy observed in this study. Notably, two of the three patients who achieved CR only received second-line medication, and all three received treatments within the first three lines of treatment. Collectively, based on the high-level evidence from the DB series of trials combined with the subgroup data from this study, T-DXd has certain clinical potential in the treatment of HER2-positive BC with BM. The potential relationship between its intracranial efficacy and treatment timing observed in our study merits validation in future large-scale, prospective studies.

### 4.4 Limitations

This single-center, retrospective, real-world investigation, aims to objectively evaluate the efficacy and safety of T-DXd in patients with HER2-positive or HER2-low expression ABC. Despite its contributions, a few limitations should be aware of. First, due to the relatively short follow-up duration at the data cutoff, not enough deaths were observed, and overall survival (OS) data were immature and could not be meaningfully analyzed. This also may compromise the accuracy of the reported mPFS and result in insufficient evaluation of long-term safety events. Second, the limited sample size may reduce statistical power and be insufficient to adequately estimate the incidence of low-frequency AEs, particularly ILD, making it challenging to accurately reflect the real-world efficacy and safety of T-DXd in this population. Furthermore, the single-center design may introduce geographical specificity in the baseline characteristics of enrolled patients, which could differ from those of real-world populations in other regions, further limiting the external validity of the findings. Future large-scale, multicenter prospective studies are warranted for further validation. In order to give more solid evidence for confirming the practical effectiveness and safety of T-DXd as well as for supporting accurate and personalized treatment for patients, research should also concentrate on enhancing long-term survival statistics and bolstering AEs surveillance during treatment.

## 5 Conclusion

This retrospective real-world study evaluated the efficacy and safety of T-DXd in 104 Chinese patients with ABC, including HER2-positive and HER2-low expression subtypes. The results were consistent with those from the DB series of clinical trials. Critically, subgroup analyses indicated that patients with HER2-positive disease and BM derived significant clinical benefit, supporting the early use of T-DXd in the treatment sequence for this population. This study bridges the gap between clinical trial evidence and real-world application, validating the practical value of T-DXd across diverse clinical scenarios.

## Data Availability

The raw data supporting the conclusions of this article will be made available by the authors, without undue reservation.
